# Studies of Circuit Design, Structural, Relaxation and Potential Stability of Polymer Blend Electrolyte Membranes Based on PVA:MC Impregnated with NH_4_I Salt

**DOI:** 10.3390/membranes12030284

**Published:** 2022-02-28

**Authors:** Muaffaq M. Nofal, Shujahadeen B. Aziz, Mohamad A. Brza, Sozan N. Abdullah, Elham M. A. Dannoun, Jihad M. Hadi, Ary R. Murad, Sameerah I. Al-Saeedi, Mohd F. Z. Kadir

**Affiliations:** 1 Department of Mathematics and General Sciences, Prince Sultan University, P.O. Box 66833, Riyadh 11586, Saudi Arabia; muaffaqnofal69@gmail.com; 2 Hameed Majid Advanced Polymeric Materials Research Lab., Physics Department, College of Science, University of Sulaimani, Qlyasan Street, Sulaimani 46001, Iraq; 3 Department of Civil Engineering, College of Engineering, Komar University of Science and Technology, Sulaimani 46001, Iraq; 4 Medical Physics Department, College of Medicals & Applied Science, Charmo University, Sulaimani 46023, Iraq; mohamad.brza@gmail.com; 5 Department of Chemistry, College of Science, University of Sulaimani, Qlyasan Street, Sulaimani 46001, Iraq; sozan.abdulla@univsul.edu.iq; 6 General Science Department, Woman Campus, Prince Sultan University, P.O. Box 66833, Riyadh 11586, Saudi Arabia; elhamdannoun1977@gmail.com; 7 Department of Medical Laboratory of Science, College of Health Sciences, University of Human Development, Sulaimani 46001, Iraq; jihad.chemist@gmail.com; 8 Department of Pharmaceutical Chemistry, College of Medical and Applied Sciences, Charmo University, Sulaimani 46023, Iraq; ary.murad@charmouniversity.org; 9 Department of Chemistry, College of Science, Princess Nourah Bint Abdulrahman University, Riyadh 11671, Saudi Arabia; sialsaeedi@pnu.edu.sa; 10 Centre for Foundation Studies in Science, University of Malaya, Kuala Lumpur 50603, Malaysia; mfzkadir@um.edu.my

**Keywords:** polymer electrolyte, FTIR, circuit modeling, TNM and LSV, impedance, dielectric properties, relaxation process

## Abstract

This work presents the fabrication of polymer electrolyte membranes (PEMs) that are made of polyvinyl alcohol-methylcellulose (PVA-MC) doped with various amounts of ammonium iodide (NH_4_I). The structural and electrical properties of the polymer blend electrolyte were performed via the acquisition of Fourier Transform Infrared (FTIR) and electrical impedance spectroscopy (EIS), respectively. The interaction among the components of the electrolyte was confirmed via the FTIR approach. Electrical impedance spectroscopy (EIS) showed that the whole conductivity of complexes of PVA-MC was increased beyond the addition of NH_4_I. The application of EEC modeling on experimental data of EIS was helpful to calculate the ion transport parameters and detect the circuit elements of the films. The sample containing 40 wt.% of NH_4_I salt exhibited maximum ionic conductivity (7.01 × 10^−8^) S cm^−1^ at room temperature. The conductivity behaviors were further emphasized from the dielectric study. The dielectric constant, ε’ and loss, ε’’ values were recorded at high values within the low-frequency region. The peak appearance of the dielectric relaxation analysis verified the non-Debye type of relaxation mechanism was clarified via the peak appearance of the dielectric relaxation. For further confirmation, the transference number measurement (TNM) of the PVA-MC-NH_4_I electrolyte was analyzed in which ions were primarily entities for the charge transfer process. The linear sweep voltammetry (LSV) shows a relatively electrochemically stable electrolyte where the voltage was swept linearly up to 1.6 V. Finally, the sample with maximum conductivity, ion dominance of *t_ion_* and relatively wide breakdown voltage were found to be 0.88 and 1.6 V, respectively. As the ions are the majority charge carrier, this polymer electrolyte could be considered as a promising candidate to be used in electrochemical energy storage devices for example electrochemical double-layer capacitor (EDLC) device.

## 1. Introduction

In the near future, there is predicted to be a rapid increase in the need for energy storage devices like supercapacitors, fuel cells, portable electronics, batteries, sensors, etc. To meet this requirement, an alternative component as a major function of energy storage and energy production has been intensively studied [1,2,3]. Polymer electrolytes (PEs) have been intensively investigated due to their unique applications and their importance in theoretical studies in domains of energy storage and electrochemistry [4]. Solid state coordinated compounds in the form of polymer electrolytes with improved ionic conductivity can be utilized as solid membranes [5]. Focus on solid polymer electrolytes extensively and intensively have been looked at since the outstanding work carried out by Wright et al. and Armand et al. [6]. Polymer electrolytes (PEs) are made up of alkali metal salts with a low dissociation energy that are dissolved in polar polymers and used in electrochemical devices [7]. Bio-based polymers (BBPs) have increased in popularity as powerful alternatives to conventional polymers as a result of a significant amount of research. This is due to the wide utilization of these materials in electrochemical devices as ways of solving global concerns. These bio-based polymers can be extracted naturally from living organisms [8]. There is agreement that polymer electrolytes with the appropriate conductivity should be developed for use as separators in electrochemical devices because of having relatively high ionic conductivity as intrinsic properties of SPEs [9,10].

Two forms of polyvinyl alcohol (PVA) polymer have been familiarized with; pure polymer [11,12,13] and blended polymer containing polyvinyl pyrrolidone [14], arginine [15] and carboxymethyl cellulose [16]. To modify the properties of these classes of electrolytes, PVA is one of the suitable polymers, having the capability to receive modifications. Moreover, PVA possesses desired properties, for instance, sufficient charge storing capability, satisfactory strength, non-toxicity, and semi-crystalline [17,18,19]. Biopolymers are natural polymers that can be available in various kinds of sources. Biopolymers are often affordable, abundant in nature, have good solvent compatibility, and are very stable when creating a film [20,21,22]. A number of studies on biopolymers, e.g., carrageenan, chitosan (CS), gelatin, chitin, dextran, starch, and cellulose have all been identified as polymer hosts with ionic conductivity ranging from 10^−5^ to 10^−3^ S cm^−1^ [23,24,25].

Herein, methylcellulose (MC) is an example of a natural biopolymer that consists of a methyl substituent that is attached to a linear chain of glucose via β-(1,4)-glycosidic bond [26]. This type of polymer enriches oxygen atoms that contain lone pairs of electrons; thereby, it has enough polarity [27]. In other words, MC possesses amphiphile property as a consequence of existing two heads: hydrophobic polysaccharide and hydrophilic carboxylic functional group [28].

Nowadays, polymer blends have been under intensive investigation. This can be correlated to the possibility of modifying polymer materials by forming polymer blends. It is important to notice that the properties of the blended polymers are not only different from individual components, but are also much better [29]. This kind of polymer is a mixture that is physically mixed with characteristic structures. These physically mixed polymers contain secondary forces; in other words, there is no existence of covalent bonding where the components of the blended polymers are in interaction at the molecular level [30]. In previous work, it has been confirmed that improvement in conductivity of a polymer electrolyte is possible if the host polymer is blended with another one. The blending process of polymers can provide structural stability [29].

In this field, the usage of single MC-based electrolytes has been reported [31,32,33]. Blending (mixing) potato starch [34], maize starch [35], and chitosan [36] have been documented. The MC possesses several desired properties, for instance, biocompatibility, thermal stability, mechanical strength, and non-toxicity [37]. To be utilized in electrochemical energy devices, the conductivity of a PE has to be achieved first in order to decide whether it is eligible or not. The conductivity of the polymer electrolyte can successfully be improved via either involvement of salt or plasticizer [38,39]. For this purpose, lithium ions have been added because of their small size; however, the ion itself is unsafe to surroundings after releasing because of its non-biodegradability and also it is one of the expensive ions [40]. Shamsuri et. al., [41] fabricated polymer blend electrolyte systems based on PVA-MC doped with various quantities of ammonium thiocyanate (NH_4_SCN). They discovered that blending PVA and MC polymers and ammonium salts improved the ionic conductivity by up to 10^−4^ S/cm. In SPEs as a field of condensed matter physics, both the charge transport process and ion relaxation are the most intense subjects [42]. Many studies have shown that ammonium salts have good polymer electrolyte characteristics with increased ion dissociation, in addition to decreasing environmental pollution caused by the use of lithium salts. Furthermore, because of their ability to attain high ionic conductivity while maintaining good compatibility and thermal stability, ammonium salts are commonly used in the development of polymer electrolyte systems. Additionally, the lattice energy of NH_4_I is 605.3 kJ/mol, showing a significant degree of salt dissociation into ions [43,44,45,46]. Buraidah and Arof [47] have employed ammonium iodide (NH_4_I) as an electrolyte, claiming that it increased ionic conductivity over other ammonium salts. Based on the transference number measurement (TNM) result, ions are the majority charge carrier in the polymer electrolyte, which confirms its application in electrochemical energy storage devices, for example, electrochemical double-layer capacitor (EDLC) devices.

The purpose of this work is to use AC impedance spectroscopy to investigate the conductivity and relaxation mechanisms involved with ion transport. The electrical and dielectric characteristics of materials are studied using this technique. In addition, the investigation of the ion transport process and relaxation process in PVA:MC electrolytes has been prioritized.

## 2. Materials and Methods

### 2.1. Sample Preparation

Poly (vinyl) alcohol (PVA)and methylcellulose (MC) with average molecular weights of 35,000 g/mol and 4000 cP, respectively, were provided by Sigma Aldrich and used as raw materials. Ammonium iodide (NH_4_I) salt was used as an H^+^ ion provide provider. The above raw materials were used to synthesize PVA:MC:NH_4_I polymer electrolyte samples using the solution casting method. For this purpose, 80 wt.% of PVA polymer was dissolved in 30 mL of distilled water (DW) at 80 °C. Then, 20 wt.% of MC polymer was dissolved in 30 mL of DW at room temperature (RT) for 3 h. The PVA solution was cooled down to RT. Then, MC and PVA polymers solutions were combined with a magnetic stirrer. After that, 10 to 50 wt.% in step of 10 of NH_4_I was poured to the PVA:MC solution and stirred constantly to prepare PVA:MC:NH_4_I. The samples with (10, 20, 30, 40, and 50) wt.% NH_4_I were coded as PMCVE1, PMCVE2, PMCVE3, PMCVE4, and PMCVE5, respectively. Finally, the solutions of polymer electrolytes were inserted into Petri dishes and then left to evaporate regularly at RT to fabricate PVA:CS:NH_4_I blend SPE film.

### 2.2. Measurements

The impedance of the films was measured using electrical impedance spectroscopy (EIS) using HIOKI 3532-50 LCR HiTESTER at the frequency between 50 Hz and 5000 kHz at RT. The films were inserted between two stainless steel (SS) electrodes and then the impedance of the samples was measured. The ionic conductivity and dielectric properties of the films were measured using the EIS method. Equation (1) was used in measuring ionic conductivity:

(1) $$\sigma_{dc}=\left(\frac{1}{R_{b}}\right)\times \left(\frac{t}{A}\right)$$

where; *t* is the thickness and *A* is the area of the films. The *R_b_* is the bulk resistance of the electrolyte, which is measured by the intersection between spike and real axis.

The linear sweep voltammetry (LSV) at a scan rate of 10 mV/s was used to measure the breakdown voltage of the film using Digi-IVY DY2300 potentiostat. The transference number measurement (TNM) for ions and electrons was measured using a digital DC power supply and V&A Instrument DP3003 at 0.2 V operating voltage at RT. Thermo Scientific/Nicolet iS10 FTIR spectrophotometer was utilized to measure the FTIR spectra of the films in the range between 4000–400 cm^−1^ with a resolution of 2 cm^−1^.

## 3. Results and Discussion

### 3.1. Impedance Analysis

Electrochemical impedance spectroscopy (EIS) has been widely employed in the study of electrochemical behavior, as well as ion transference, in a variety of ionic materials, such as electrodes and polymer electrolytes (PEs) [48,49,50,51,52].

**Figure 1:**
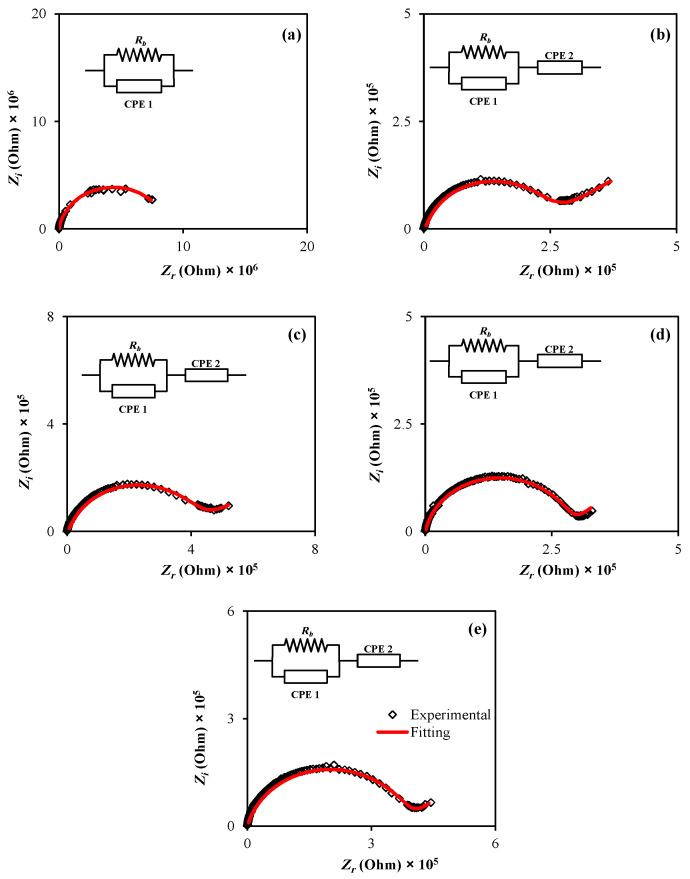
Complex plots of impedance spectra for the PVA-MC blend polymers containing (**a**) 10wt.%; (**b**) 20 wt.%; (**c**) 30 wt.%; (**d**) 40 wt.%; and (**e**) 50 wt.% of NH_4_I.

The impedance spectra for the CPE films were produced and evaluated using this approach (see Figure 1a–e). For all situations, a semicircle was formed in the high frequency area due to the CPEs bulk effect, and a tail was obtained in the low frequency zone.

Additionally, there was an incomplete semicircle at the higher frequency that is related mainly to the bulk properties (bulk resistance) of the materials. At the low frequency, there is a spike, indicating the presence of double layer capacitance at the electrode/sample interfacial region [53,54]. The establishment of the EDLC from the free charges accumulation at the electrode and electrolyte interface causes a spike (tail) in the low frequency [55]. A spike is visible in all other samples.

The electrical equivalent circuit (EEC) model has been utilized to examine the EIS, displaying the whole system under investigation [56]. The Nyquist plots for all systems may be calculated using the EEC, which includes the R_b_ for the carrier species in PE systems and two constant phase elements (CPE), as illustrated in Figure 1.

On the one hand, the connection of the constant phase element and *R_b_* in parallel is clearly seen at the high frequency. On the other hand, only constant phase element is seen at the low frequencies, supporting the formation of EDLC at the interfacial region. The constant phase elements term is often used in EEC in place of an ideal capacitor is usually used in the real system.

The Nyquist plot for the PEs was shown in terms of the EEC. It contains two constant phase elements (CPE) and *R_b_* as exhibited in the insert of Figure 1. As a result of charge buildup at the electrolyte-electrode interface, there are both constant phase elements and *R_b_* and in parallel at high frequencies and just constant phase elements at low frequencies.

The impedance of *Z_CPE_* is shown as [57,58,59]:

(2) $$Z_{CPE}=\frac{1}{C\omega^{p}}\left[\cos\left(\frac{\pi p}{2}\right)-i\sin\left(\frac{\pi p}{2}\right)\right]$$

where *C* is the capacitance of the constant phase element, *p* is the degree of deviation of the EIS plots from the vertical axis, and *ω* is the angular frequency. The EEC is represented by the real (*Z_r_*) and imaginary (*Z_i_*) components of complex impedance (Z*) (insert of Figure 1a) and the mathematical basis are shown in Equations (3) and (4):

(3) $$Z_{r}=\frac{R_{b}^{2}C_{1}\omega^{p1}\cos\left(\frac{\pi p_{1}}{2}\right)+R_{b}}{2R_{b}C_{1}\omega^{p1}\cos\left(\frac{\pi p_{1}}{2}\right)+R_{b}{}^{2}C_{1}{}^{2}\omega^{2p1}+1}+\frac{\cos\left(\frac{\pi p_{2}}{2}\right)}{C_{2}\omega^{p2}}$$

(4) $$Z_{i}=\frac{R_{b}^{2}C_{1}\omega^{p1}\sin\left(\frac{\pi p_{1}}{2}\right)}{2R_{b}C_{1}\omega^{{}^{p1}}\cos\left(\frac{\pi p_{1}}{2}\right)+R_{b}{}^{2}C_{1}{}^{2}\omega^{2P1}+1}+\frac{\sin\left(\frac{\pi p_{2}}{2}\right)}{C_{2}\omega^{p2}}$$

**Figure 2:**
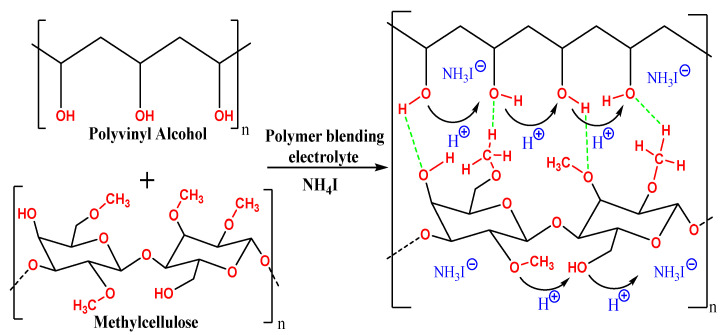
H+ conduction mechanism in PVA:MC:NH_4_I electrolyte system.

*C_*1*_* is the capacitance of the constant phase element in bulk, while *C_*2*_* is the capacitance of the constant phase element. The fitting parameters for the EEC and DC conductivity values are listed in Table 1 and Table 2, respectively. The *R_b_* is calculated by intercepting the real and spike axes [60]. Figure 2 shows polymer electrolyte structure and proposed ion conduction mechanism in the PVA:MC:NH_4_I electrolyte system.

As the impedance data is composed of a semicircle and a spike, the number density (*n*), diffusion coefficient (*D*), and mobility of ions are measured using the following relations.

The *D* is measured using Equation (5):

(5) $$D=\left(\frac{{\left(K_{2}\varepsilon_{o}\varepsilon_{r}A\right)}^{2}}{\tau_{2}}\right)$$

where *τ_*2*_* is the angular frequency reciprocal and corresponds to the minimum in *Z_i_*.

The *µ* is measured using Equation (6),

(6) $$\mu =\left(\frac{eD}{K_{b}T}\right)$$

where *T* is the absolute temperature and *k_b_* is the constant of Boltzmann.

Since conductivity (*σ_dc_*) is measured by

(7) $$\sigma_{dc}=ne\mu$$

So, the *n* is measured using Equation (8):

(8) $$n=\left(\frac{\sigma_{dc}K_{b}T\tau_{2}}{{\left(eK_{2}\varepsilon_{o}\varepsilon_{r}A\right)}^{2}}\right)$$

Based on Table 1, the *D* value is increased from 10 wt.% to 40 wt.% of the NH_4_I salt. The same trend is observed by *μ* as seen in Table 3 where μ increased. This increment is related to the increase of the flexibility of the chains of the polymer with the addition of the salt.

### 3.2. FTIR Study

**Figure 3:**
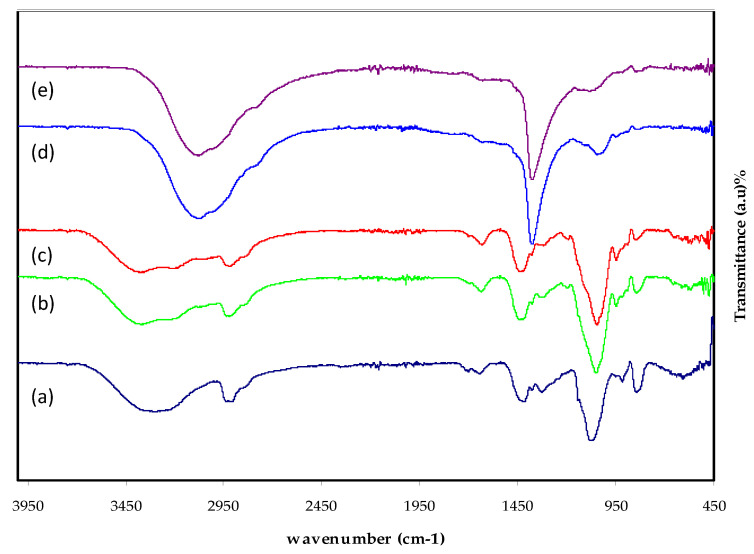
FTIR spectra illustrated the pure PVA-MC blend (0.6:0.4) and PVA:MC loaded with (**a**) 10, (**b**) 20, (**c**) 30, (**d**) 40, and (**e**) 50 wt.%: NH_4_I salt.

The composition, structure, and potential interaction between the functional groups of MC and PVA in PVA:MC blend films may all be studied using Fourier transform infrared (FTIR) spectroscopy. It is also used to look at how the blended PVA:MC interacts with the NH_4_I salt, as evidenced by changes in the location, intensity, and shape of the IR transmittance bands in the 400 to 4000 cm^−1^ range. The FTIR spectra of blended PVA:MC polymer after mixing with various weight percent of NH_4_I are shown in Figure 3 within the specified range.

It is evidenced that there are interactions between the salt and the polymer matrix, creating a complex system. It is seen that when the interaction occurs between the PVA:MC and ammonium iodide, the peaks disappear. This verifies the complexation of the macromolecular salt successfully. The C=O, CH_2_, C-H, C-O, and O-H bonds have characteristic bands for MC and PVA [50]. Therefore, any change is considered as evidence of the compatibility between the components of the system. The increase of the amorphous structure in the blend of PVA:MC is another element of the salt’s entry into the polymer matrix. The vibrational peak at a wavenumber of ~3000 cm^−1^ correlates to the O-H stretching. From Figure 3, it is seen that the OH bands become broader accompanied by intensity attenuation of the peaks for the PVA:MC system, indicating an extent of crystallinity [61,62,63].

There is a shift in the hydroxyl group’s (-OH) absorption frequency as a result of the complex development between the NH_4_I and the host polymer. The stretching band at 1709 cm^−1^ is connected to the C=O stretching of carboxylic groups.

The carboxylic groups in the films can create intramolecular or intermolecular hydrogen bonds with either OH groups or carboxylic groups [64]. Complete complexation among the salt cations and polymer functional groups is indicated by a reduction in the intensity of transmittance and a shift in band position. The vibration within the polar group decreases as a consequence of the electrostatic interaction between the functional group and the salt cation [64,65]. Shifting in peak position primarily indicates that the state of electron distribution or hybridization in the molecular bond has changed. Attenuation in the peak intensity often shows that the number of functional groups associated with the molecular bond (per unit volume) decreases [66].

The bands that appear at the wavenumber of ~2900 cm^−1^ correspond to the (C-H) stretching, while they disappeared at the high content of added salt. Additionally, the bands located at ~1650 for the PMCVE1, PMCVE2, and PMCVE3 are due to (C=O) stretching. It is noticeable that the intensity of the bands of (C=O) stretching is significantly decreased in the PMCVE3, and PMCVE5 samples, respectively.

### 3.3. Dielectric Properties

It is well-known that the dielectric constant is one of the best indicators for studying and evaluating the conductivity of polymer electrolytes [67]. Both axes of the spectrum are indicators of specific properties.

The dielectric constant (ε′) determines polarization or dipole alignment is proportional to capacitance, whereas dielectric loss (ε″) represents dielectric loss linked to conductance and reflects the amount of energy required for dipole alignment [68]. The finding of the growth of ion pair creation from the aggregation of dissolved ion pairs is fundamentally and technologically significant since such formations might impair electrical conductivity [69].

Just recall that Equations (9) and (10) are used to determine both the ε′ and ε″ of the dielectric permittivity.

(9) $$\varepsilon_{r}=\frac{Z_{i}}{\omega \;C_{o}(Z_{r}{}^{2}+Z_{i}{}^{2})}$$

(10) $$\varepsilon_{i}=\frac{Z_{r}}{\omega C_{o}(Z_{r}{}^{2}+Z_{i}{}^{2})}$$

Here, *C*_o_ is the vacuum capacitance and equal to ε_o_A/t (where *t* and *A* are the thickness and area of the sample, respectively), and ω is the angular frequency and equal to = 2π*f*.

**Figure 4:**
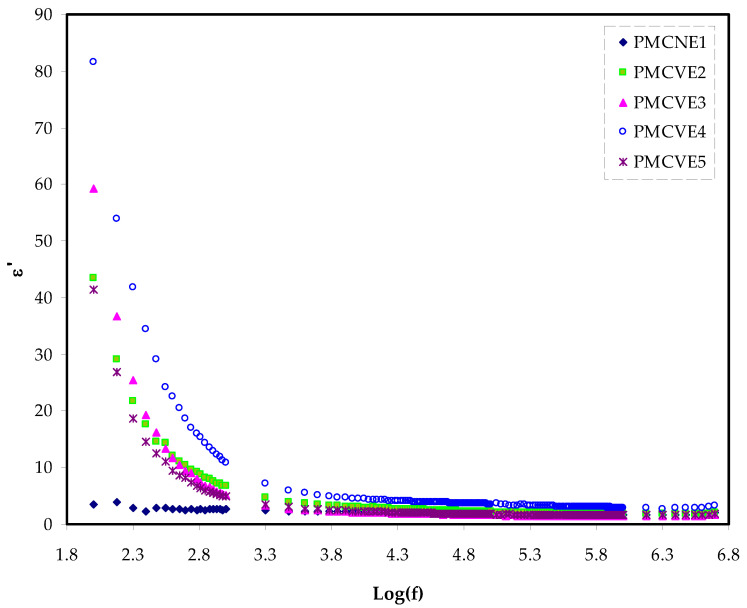
Dielectric plot for ε′ variation against frequency for the MCKI samples.

**Figure 5:**
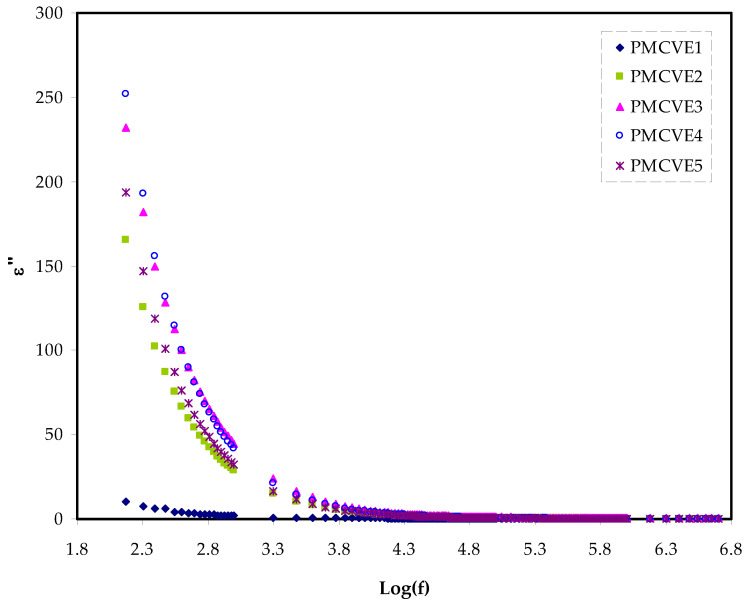
Dielectric plot for ε″ variation against frequency for the PMCVE samples.

At low frequency, both the ε′ and ε″ are relatively high, as shown in Figure 4 and Figure 5, as is likely to occur for the blend electrolyte samples.

The polarization of space charge or charge buildup at the electrode-electrolyte interface can be linked to these high reported values of two parameters [70].

In contrast, at the high frequency, the values are relatively low because of the responsibility of the bulk property. It is worth noting that decreasing the frequency of applied electric field (EF) lengthens the charge carrier’s available drift time.

This results in increasing the values of the dielectric constant and dielectric loss as well [66]. At the high-frequency region, a quick periodic reversal of the EF occurs, resulting in no allowance of excess ions diffusion in the EF direction. Additionally, polarization decreases as a consequence of the charge accumulation at the interfacial region; thereby, shrinking occurs and becomes frequency independent [71]. At the 40 wt.% of NH_4_I insertion into the polymer matrices, the maximum dielectric constant value was recorded. The fact that amorphous areas predominate in the system can be linked to the growth of the ε′ and ε″ [72,73].

More attractive notice is the relatively high value of dielectric loss compared to the value of ε′, as clearly seen in Figure 4 and Figure 5. This is due to two variables, dielectric polarization processes and DC conduction, both contributing to increasing dielectric loss [72].

### 3.4. Tangent Delta Analysis

The dissipation factor is defined as the loss tangent (tan δ). It is the energy loss to energy store ratio in a periodic field that may be calculated using Equation (4).

**Figure 6:**
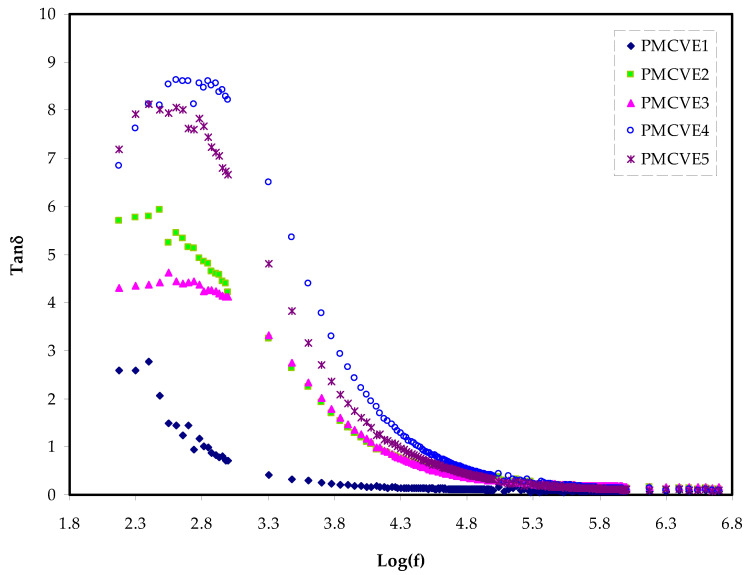
The loss tangent change with frequency for the PMCVE SPEs.

In order to comprehend the relaxation of dipoles in polymer electrolytes, it is critical to define dielectric relaxation. As the concentration of NH_4_I is raised up to 40%, the maximum tangent is likely to move to the higher frequency area (Figure 6).

In polymer electrolytes with high electrical conductivity, the polarization of charge carriers in the materials causes relaxation, which leads to the disappearance of dielectric relaxation peaks caused by induced dipole or permanent dipole.

Figure 6 illustrates the loss tangent variation over a range of frequencies for various solid polymer electrolytes. It is obviously seen that the single relaxation peak is considered as a fingerprint of ionic conduction throughout the polymer body via segmental motion of the chains [74,75]. It is of great importance to interpret the loss tangent shape on the basis of the model of Koops phenomenological. However, in the homogeneous systems, the low frequency dispersion curve negative slope indicates that loss is dominated by conduction at low frequency within a parallel RC circuit. As the frequency is raised, the loss tangent rises, indicating a maximum at a certain frequency as a result of a fast ascend in the active component (ohmic) relative to the reactive component (capacitive). This indicates that the current’s active component is fundamentally and efficiently working [76,77]. At high frequency, the loss tangent decreases as frequency increases.

The active component of the current is frequency independent, while the reactive component is directly proportional to frequency. As a result of the presence of several non-Debye relaxation processes, the loss tangent peaks become broader.

These interpretations are based on EEC fitting of experimentally obtained impedance data that shows a shift of the peak to the higher frequency. It is also deduced that a decrease in the relaxation time is ascribed to carrier mobility increasing. The bond breakdown originating from the dipoles is reflected by a boost in band intensity [75,76].

The mobility, carrier density, and diffusion coefficient are three key factors for evaluating the ion transport phenomena, as previously stated. Finally, utilizing the single relaxation peak of tanδ spectra as shown in Figure 6, the relaxation time (τ = 1⁄2πf_max_) may be determined [78].

The electric modulus has been used to study the dielectric response induced by ion relaxation in which the electrode polarization effects are reduced, i.e., highlight tiny characteristics at high frequencies [79]. The following equations link the real and imaginary components of electric modulus to impedance values [80,81],

(11) $$M^{\prime }\;=\omega \;C_{o}Z_{i}$$

(12) $$M^{\prime \prime }\;=\omega \;C_{o}Z_{r}$$

**Figure 7:**
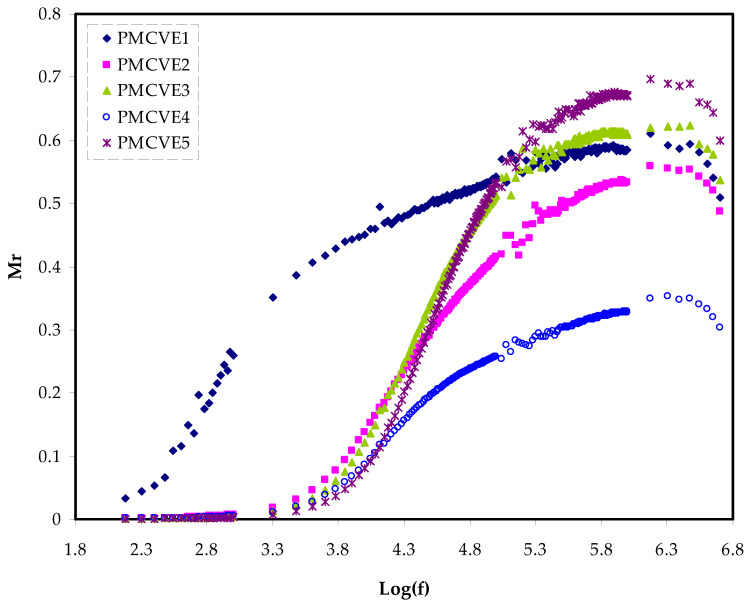
Electric modulus plot of Mr against log(f)for the PMCVE samples.

**Figure 8:**
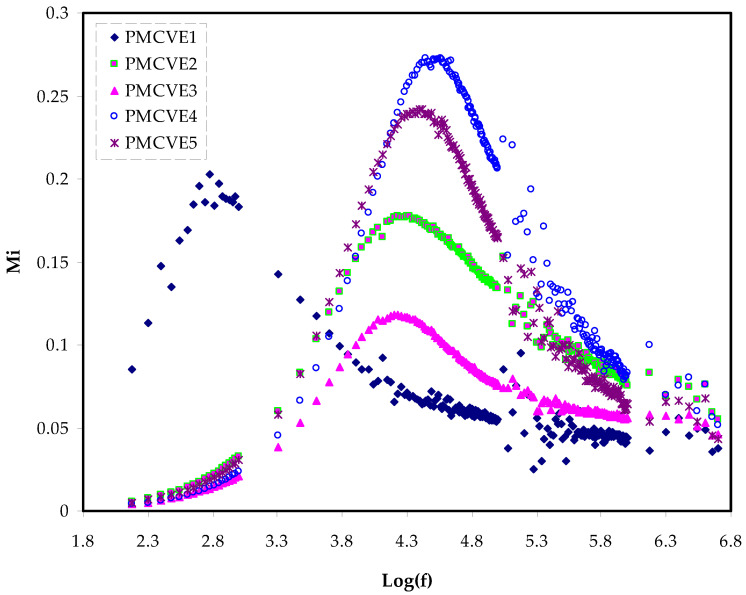
Electric modulus plot of Mi against log(f)for the PMCVE samples.

The plots of the frequency-dependence real and imaginary parts of the electrical modulus, (*M_r_* and *M_i_*), were shown in Figure 7 and Figure 8, respectively. At lower frequencies, the plot of the real component of modulus spectra shows a low value. This can be explained by the high capacitance connected with the electrodes, which promotes ion conduction migration.

The *M_r_* exhibits dispersion as the frequency is increased. This supports the samples’ non-Debye behavior [82]. Figure 8 depicts the imaginary section of modulus spectra.

The electric modules (*M*′ and *M*″) have a minimal value at low frequencies because they are reciprocals of the complex dielectric constant.

The use of the M-formalism for studying electrical relaxation processes has recently been reported in the literature [83]. *M*″ has an asymmetrical shape, indicating that Debye’s basic exponential is insufficient to characterize the relaxation. Figure 8 shows the peaks of conductivity relaxation.

From a physics standpoint, the relaxation peak in *M*″ (Figure 8) and with no peaks in the ε″ (see Figure 5) has some significance.

It indicates that in polymer electrolytes, conduction occurs by ion charge movement across coordinated sites of the polymer, as well as segmental relaxation, that is appearance of peaks in *M*″ spectra confirm that ionic motion and polymer segmental motion are strongly coupled [84,85]. It can be seen that the relaxation peak shifted to the side of low frequency with increasing PVA. This means that when the concentration of PVA rises, so does the relaxing time. The decrease in ionic mobility is linked to an increase in relaxation time [59].

At low salt concentrations, conductivity relaxation peaks can be seen. With increasing NH_4_I concentration, the relaxation peak changed to the side of high frequency.

This means that when the concentration of NH_4_I rises the relaxation time $\left(\tau_{o}=1/\omega_{\max}\right)$ reduces.

The increase in ionic mobility in the amorphous phase of the electrolytes sample leads to a reduction in relaxation time. The electrical characteristics are well supported by the XRD findings [54].

### 3.5. Transference Number Measurements TNM

In PE systems both electrons and ions are responsible for carrying electric charges. It is critical to have a PE with a high *t_i_* and low *t_e_* in order to qualify it for use in EDLC applications. The energy storage mechanism in the EDLC is carried out by ions adsorbing and desorbing on the surface of carbon electrons at a specific interface area. After exposing the samples to an operating voltage of 0.8 V, the TNM analysis for the PE was conducted.

**Figure 9:**
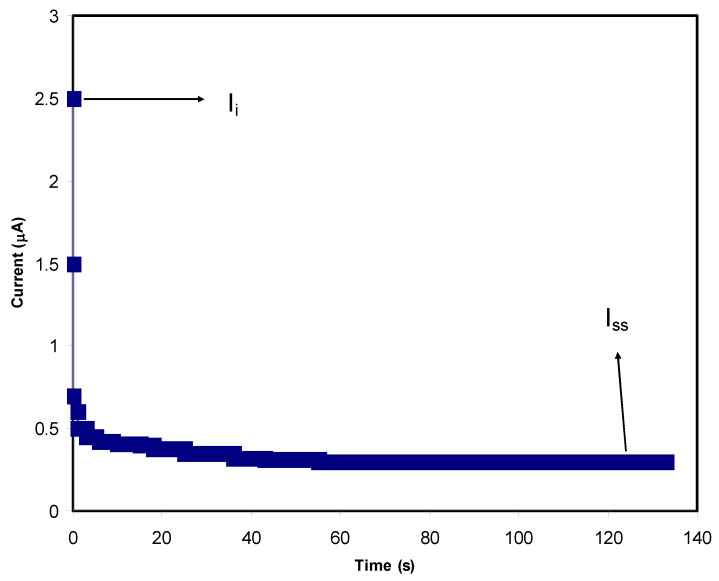
The polarization curve of current against time for the PVC-MC polymer containing 40 wt.% of salt.

**Figure 10:**
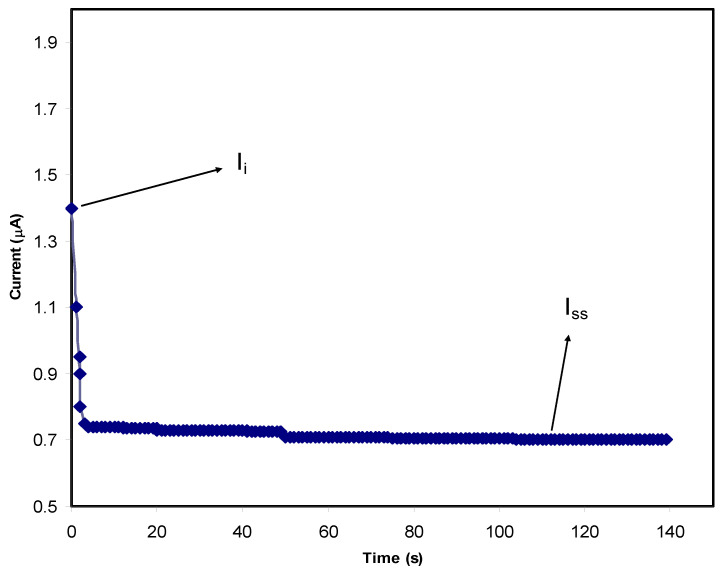
The polarization curve of current against time for the PVC-MC polymer containing 50 wt.% of salt.

Figure 9 and Figure 10 show the response of polarization within the PVA-MC blend polymer containing 40 wt.% and 50 wt.% NH_4_I, respectively. After the perturbation of the systems by applying charge transfer occurs via ion and electrons together towards the electrodes resulting in a high *I_i_* of 2.5 µA for 40 wt.% and 1.4 µA for 50 wt.% of the salt. As time lasts, ion movement is blocked at the surface of SS, causing current flow lowering. It is important to notice that a current plateau is recorded beyond 50 s at 0.25 µA for 40 wt.% and 0.7 µA for 50 wt.%. This state is the so called steady-state in which the PE is now is completely under polarization. Only electrons can pass through the SS electrode in the steady-state. This is a property of an ionic conductor that occurs when electrons are transferred [86]. From the following equations, *t_i_* and *t_e_* can be obtained:

(13) $$t_{ion}=\frac{I_{i}-I_{SS}}{I_{i}}$$

(14) $$t_{ion}=1-t_{el}$$

here, *t_ion_* and *t_el_* indicate the symbols for ion transport and electron transfer, respectively, *I_i_* indicates the initial current, which comprises both electrons and ions, and *I_ss_* means the steady-state current, which only covers electrons. It was seen that t_ion_ is 0.88 for 40 wt.% of NH_4_I while it drops to 0.56 for 50 wt.% of the salt. The values for *t_i_* and *t_e_* are 0.964 and 0.036, respectively. For Mg(CH_3_COO)_2_, Mg(NO_3_)_2_, and MgCl_2_, the *t_i_* is 0.95 [87,88,89].

### 3.6. Linear Sweep Voltammetry (LSV)

**Figure 11:**
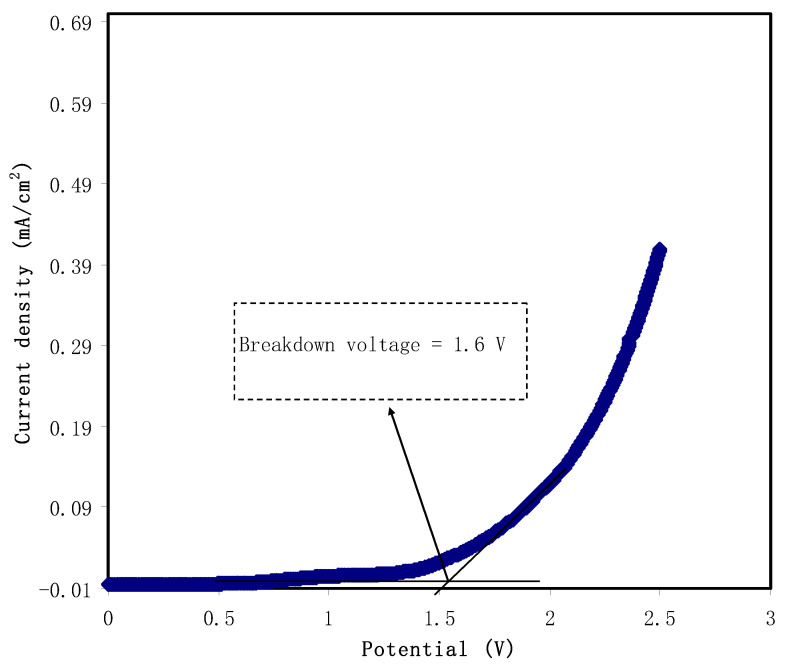
The LSV plot for the highest conducting sample.

Linear sweep voltammetry (LSV) recording is useful in determining the potential stability of the PE which was carried out at a 20 mVs^−1^ sweep rate. The potential stability of the PE is necessary technologically. This is because of the rapid charge-discharge process in energy devices, leading to degrading of the PE. It is imperative to have a wide potential wide of PE in order to avoid decomposition during operation. It is straightforward to record LSV for the PE that will be utilized in the supercapacitors, fuel cells, solar cells and batteries [90,91]. Figure 11 shows the recorded potential-current profile that swept linearly up to 3.5 V. It is noted that the PE is stable at potential <1.6 V, which is determined from the huge current rise at this potential.

As the voltage is changed from 2.1 to 2.5 V, the dramatic current increases from 0.01 to 0.41 mA cm^−2^, which can be observed more clearly. To put it another way, the huge current increase implies that the polymer is degrading [92].

## 4. Conclusions

Descriptively, the fabrication of polymer electrolyte (PE) comprised a blending process of the solid polymer electrolytes (SPEs) polyvinyl alcohol- methylcellulose (PVA-MC) loaded with various quantities of ammonium iodide (NH_4_I). Fourier Transform Infrared (FTIR) study confirmed a strong interaction between the electrolyte components of polymer matrix PVA-MC and the ionic dopant (NH_4_^+^ and I^−^). It has been proved that there is an effective complexation and compatibility between the components of PE. The blending can be confirmed from the peak shifting and peak intensity attenuation via the FTIR test. It is concluded that the structural disorder is caused effectively by the addition of NH_4_I. It is also deduced that the substantial rise in ionic conductivity values resulted from the salt addition. The highest conductivity of 7.01 × 10^−8^ S cm^−1^ was measured for the sample loaded with 40 wt.% NH_4_I. The EEC modeling on experimental data of EIS was helpful to calculate the ion transport parameters and detect the circuit elements of the films. The transport parameters of *μ*, *n*, and *D* were increased with the salt increment till 40 wt.% of NH_4_I. The trend of DC conductivity was described with the help of dielectric properties. The highest conducting electrolyte displays the ion dominancy where the *t_ion_* is 0.88. It was shown by the LSV that the potential stability of the electrolyte is 1.6 V.

## Figures and Tables

**Table 1:** The values of the circuit elements for the PMCVE electrolyte systems.

Sample	p1 (rad)	p2 (rad)	CPE1 (F^−1^)	CPE2 (F^−1^)
PMCVE1	0.92		1.18 × 10^−10^	
PMCVE2	0.91	0.40	1.33 × 10^−10^	4.08 × 10^−7^
PMCVE3	0.86	0.38	1.61 × 10^−10^	4.55 × 10^−7^
PMCVE4	0.90	0.52	1.67 × 10^−10^	5.00 × 10^−7^
PMCVE5	0.86	0.48	1.69 × 10^−10^	5.56 × 10^−7^

**Table 2:** Ionic conductivity and bulk resistance values for the PMCVE electrolyte systems.

Sample	R_b_ (Ω)	σ_dc_ (S/cm)
PMCVE1	8.80 × 10^6^	1.75 × 10^−9^
PMCVE2	2.20 × 10^5^	7.01 × 10^−8^
PMCVE3	3.90 × 10^5^	3.95 × 10^−8^
PMCVE4	2.80 × 10^5^	5.51 × 10^−8^
PMCVE5	3.80 × 10^5^	4.06 × 10^−8^

**Table 3:** The values of the ionic transport parameters for the PMCVE electrolyte systems.

Sample	D (cm^2^ s^−1^)	µ (cm^2^ V^−1^ s)	n (cm^−3^)
PMCVE1			
PMCVE2	3.55 × 10^−9^	1.38 × 10^−7^	3.16 × 10^18^
PMCVE3	9.41 × 10^−10^	3.67 × 10^−8^	6.73 × 10^18^
PMCVE4	1.18 × 10^−9^	4.61 × 10^−8^	7.45 × 10^18^
PMCVE5	3.17 × 10^−10^	1.24 × 10^−8^	2.05 × 10^19^

## Data Availability

The data presented in this study are available on request from the corresponding author.

## References

[1] Aziz S.B., Dannoun E.M., Murad A.R., Mahmoud K.H., Brza M., Nofal M.M., Elsayed K.A., Abdullah S.N., Hadi J.M., Kadir M. (2021). Influence of scan rate on CV Pattern: Electrical and electrochemical properties of plasticized Methylcellulose: Dextran (MC:Dex) proton conducting polymer electrolytes. Alex. Eng. J..

[2] Shaari N., Kamarudin S.K. (2019). Recent advances in additive-enhanced polymer electrolyte membrane properties in fuel cell applications: An overview. Int. J. Energy Res..

[3] Shaari N., Kamarudin S.K., Bahru R. (2020). Carbon and graphene quantum dots in fuel cell application: An overview. Int. J. Energy Res..

[4] Aziz S.B., Ali F., Anuar H., Ahamad T., Kareem W.O., Brza M., Kadir M., Abu Ali O.A., Saleh D.I., Asnawi A. (2021). Structural and electrochemical studies of proton conducting biopolymer blend electrolytes based on MC:Dextran for EDLC device application with high energy density. Alex. Eng. J..

[5] Aziz S.B., Abidin Z.H.Z., Arof A.K. (2010). Influence of silver ion reduction on electrical modulus parameters of solid polymer electrolyte based on chitosan-silver triflate electrolyte membrane. Express Polym. Lett..

[6] Pawlicka A., Danczuk M., Wieczorek W., Zygadlo-Monikowska E. (2008). Influence of Plasticizer Type on the Properties of Polymer Electrolytes Based on Chitosan. J. Phys. Chem. A.

[7] Aziz S.B., Kadir M.F.Z., Abidin Z.H.Z. (2016). Structural, Morphological and Electrochemical Impedance Study of CS:LiTf based Solid Polymer Electrolyte: Reformulated Arrhenius Equation for Ion Transport Study. Int. J. Electrochem. Sci..

[8] Franceschi E., De Cezaro A., Ferreira S.R.S., Kunita M.H., Muniz E., Rubira A., Oliveira J.V. (2011). Co-Precipitation of Beta-Carotene and Bio-Polymer Using Supercritical Carbon Dioxide as Antisolvent. Open Chem. Eng. J..

[9] Wang Y., Song Y., Xia Y. (2016). Electrochemical capacitors: Mechanism, materials, systems, characterization and applications. Chem. Soc. Rev..

[10] Hadi J.M., Aziz S.B., Mustafa M.S., Brza M.A., Hamsan M.H., Kadir M.F.Z., Ghareeb H.O., Hussein S.A. (2020). Electrochemical Impedance study of Proton Conducting Polymer Electrolytes based on PVC Doped with Thiocyanate and Plasticized with Glycerol. Int. J. Electrochem. Sci..

[11] Lim C.-S., Teoh K.H., Liew C.-W., Ramesh S. (2014). Capacitive behavior studies on electrical double layer capacitor using poly (vinyl alcohol)–lithium perchlorate based polymer electrolyte incorporated with TiO_2_. Mater. Chem. Phys..

[12] Radha K.P., Selvasekarapandian S., Karthikeyan S., Hema M., Sanjeeviraja C. (2013). Synthesis and impedance analysis of proton-conducting polymer electrolyte PVA:NH4F. Ionics.

[13] Hema M., Selvasekerapandian S., Hirankumar G., Sakunthala A., Arunkumar D., Nithya H. (2009). Structural and thermal studies of PVA:NH4I. J. Phys. Chem. Solids.

[14] Sundaramahalingam K., Muthuvinayagam M., Nallamuthu N., Vanitha D., Vahini M. (2019). Investigations on lithium acetate-doped PVA/PVP solid polymer blend electrolytes. Polym. Bull..

[15] Bhuvaneswari R., Begam M.R., Karthikeyan S., Selvasekarapandian S. (2019). Development and characterization of proton conducting polymer electrolyte based on PVA:Arginine: NH4SCN. AIP Conf. Proc..

[16] Mazuki N., Majeed A.A., Nagao Y., Samsudin A. (2019). Studies on ionics conduction properties of modification CMC-PVA based polymer blend electrolytes via impedance approach. Polym. Test..

[17] Liew C.-W., Arifin K., Kawamura J., Iwai Y., Ramesh S., Arof A. (2015). Electrical and structural studies of ionic liquid-based poly(vinyl alcohol) proton conductors. J. Non-Cryst. Solids.

[18] Liew C.-W., Ramesh S., Arof A.K. (2014). Investigation of ionic liquid-based poly(vinyl alcohol) proton conductor for electrochemical double-layer capacitor. High Perform. Polym..

[19] Aziz S.B., Hadi J.M., Dannoun E.M.A., Abdulwahid R.T., Saeed S.R., Marf A.S., Karim W.O., Kadir M.F. (2020). The Study of Plasticized Amorphous Biopolymer Blend Electrolytes Based on Polyvinyl Alcohol (PVA): Chitosan with High Ion Conductivity for Energy Storage Electrical Double-Layer Capacitors (EDLC) Device Application. Polymers.

[20] Hadi J.M., Aziz S.B., Nofal M.M., Hussein S.A., Hamsan M.H., Brza M.A., Abdulwahid R.T., Kadir M.F.Z., Woo H.J. (2020). Electrical, Dielectric Property and Electrochemical Performances of Plasticized Silver Ion-Conducting Chitosan-Based Polymer Nanocomposites. Membranes.

[21] Hamsan M.H., Aziz S.B., Nofal M.M., Brza M.A., Abdulwahid R.T., Hadi J.M., Karim W.O., Kadir M.F.Z. (2020). Characteristics of EDLC device fabricated from plasticized chitosan: MgCl_2_ based polymer electrolyte. J. Mater. Res. Technol..

[22] Stepniak I., Galinski M., Nowacki K., Wysokowski M., Jakubowska P., Bazhenov V.V., Leisegang T., Ehrlich H., Jesionowski T. (2016). A novel chitosan/sponge chitin origin material as a membrane for supercapacitors-preparation and characterization. RSC Adv..

[23] Hassan M.F., Azimi N.S.N., Kamarudin K.H., Sheng C.K. (2018). Solid polymer electrolytes based on starch-Magnesium Sulphate: Study on morphology and electrical conductivity. ASM Sci. J..

[24] Sudhakar Y.N., Selvakumar M., Bhat D.K. (2015). Preparation and characterization of phosphoric acid-doped hydroxyethyl cellulose electrolyte for use in supercapacitor. Mater. Renew. Sustain. Energy.

[25] Moniha V., Alagar M., Selvasekarapandian S., Sundaresan B., Hemalatha R., Boopathi G. (2018). Synthesis and characterization of bio-polymer electrolyte based on iota-carrageenan with ammonium thiocyanate and its applications. J. Solid State Electrochem..

[26] García M.A., Pinotti A., Martino M., Zaritzky N. (2009). Electrically treated composite FILMS based on chitosan and methylcellulose blends. Food Hydrocoll..

[27] Shuhaimi N.E.A., Alias N.A., Kufian M.Z., Majid S.R., Arof A.K. (2010). Characteristics of methyl cellu-lose-NH_4_NO_3_-PEG electrolyte and application in fuel cells. J. Solid State Electrochem..

[28] Samsudin A.S., Kuan E.C.H., Isa M.I.N. (2011). Investigation of the potential of proton-conducting biopolymer elec-trolytes based methyl cellulose-glycolic acid. Int. J. Polym. Anal. Charact..

[29] Kadir M., Aspanut Z., Majid S., Arof A. (2010). FTIR studies of plasticized poly(vinyl alcohol)–chitosan blend doped with NH_4_NO_3_ polymer electrolyte membrane. Spectrochim. Acta Part A Mol. Biomol. Spectrosc..

[30] Sudhakar Y.N., Selvakumar M., Bhat D.K. (2012). LiClO_4_-doped plasticized chitosan and poly(ethylene glycol) blend as biodegradable polymer electrolyte for supercapacitors. Ionics.

[31] Misenan M., Khiar A. (2018). Conductivity, Dielectric And Modulus Studies of Methylcellulose-NH_4_ TF Polymer. Eurasian J. Biol. Chem. Sci. J..

[32] Salleh N.S., Aziz S.B., Aspanut Z., Kadir M.F.Z. (2016). Electrical impedance and conduction mechanism analysis of bi-opolymer electrolytes based on methyl cellulose doped with ammonium iodide. Ionics.

[33] Shuhaimi N.E.A., Teo L.P., Majid S.R., Arof A.K. (2010). Transport studies of NH_4_NO_3_ doped methyl cellulose electrolyte. Synth. Met..

[34] Yusof Y.M., Kadir M.F.Z. (2016). Electrochemical characterizations and the effect of glycerol in biopolymer electrolytes based on methylcellulose-potato starch blend. Mol. Cryst. Liq. Cryst..

[35] Abd El-Kader M.F.H., Ragab H.S. (2013). DC conductivity and dielectric properties of maize starch/methylcellulose blend films. Ionics.

[36] Misenan M.S., Isa M.I., Khiar A.S. (2018). Khiar Electrical and Structural Studies of Polymer Electrolyte based on 2 Chitosan/ Methyl Cellulose Blend Doped with BMIMTFSI. Mater. Res. Express.

[37] Ndruru S.T.C.L., Wahyuningrum D., Bundjali B., Arcana I.M. (2020). Preparation and characterization of biopolymer elec-trolyte membranes based on LiClO_4_-complexed methyl cellulose as lithium-ion battery separator. J. Eng. Technol. Sci..

[38] Ibrahim S., Yasin S.M.M., Nee N.M., Ahmad R., Johan M.R. (2011). Conductivity and dielectric behaviour of PEO-based solid nanocomposite polymer electrolytes. Solid State Commun..

[39] Kumar M., Sekhon S. (2002). Role of plasticizer’s dielectric constant on conductivity modification of PEO–NH4F polymer electrolytes. Eur. Polym. J..

[40] Gong S.-D., Huang Y., Cao H.-J., Lin Y.-H., Li Y., Tang S.-H., Wang M.-S., Li X. (2016). A green and environment-friendly gel polymer electrolyte with higher performances based on the natural matrix of lignin. J. Power Sources.

[41] Shamsuri N.A., Zaine S.N.A., Yusof Y.M., Yahya W.Z.N., Shukur M.F. (2020). Effect of ammonium thiocyanate on ionic conductivity and thermal properties of polyvinyl alcohol–methylcellulose–based polymer electrolytes. Ionics.

[42] Aziz S.B. (2013). Li+ ion conduction mechanism in poly (ε-caprolactone)-based polymer electrolyte. Iran. Polym. J..

[43] Aziz S.B., Nofal M.M., Kadir M.F.Z., Dannoun E.M.A., Brza M.A., Hadi J.M., Abdulla R.M. (2021). Bio-Based Plasticized PVA Based Polymer Blend Electrolytes and Electrochemical Properties. Materials.

[44] Hema M., Selvasekerapandian S., Sakunthala A., Arunkumar D., Nithya H. (2008). Structural, vibrational and electrical characterization of PVA–NH_4_Br polymer electrolyte system. Phys. B Condens. Matter.

[45] Hema M., Selvasekarapandian S., Arunkumar D., Sakunthala A., Nithya H. (2008). FTIR, XRD and ac impedance spectroscopic study on PVA based polymer electrolyte doped with NH_4_X (X=Cl, Br, I). J. Non-Cryst. Solids.

[46] Yusof Y.M., Majid N.A., Kasmani R.M., Illias H.A., Kadir M.F.Z. (2014). The Effect of Plasticization on Conductivity and Other Properties of Starch/Chitosan Blend Biopolymer Electrolyte Incorporated with Ammonium Iodide. Mol. Cryst. Liq. Cryst..

[47] Buraidah M.H., Arof A.K. (2011). Characterization of chitosan/PVA blended electrolyte doped with NH4I. J. Non-Cryst. Solids.

[48] Cho S., Chen C.-F., Mukherjee P.P. (2015). Influence of Microstructure on Impedance Response in Intercalation Electrodes. J. Electrochem. Soc..

[49] Mustafa M.S., Ghareeb H.O., Aziz S.B., Brza M.A., Al-Zangana S., Hadi J.M., Kadir M.F.Z. (2020). Electrochemical Characteristics of Glycerolized PEO-Based Polymer Electrolytes. Membranes.

[50] Aziz S.B., Brza M.A., Hamsan E.M.A.D.M.H., Hadi J.M., Kadir M.F.Z., Abdulwahid R.T. (2020). The Study of Electrical and Electrochemical Properties of Magnesium Ion Conducting CS: PVA Based Polymer Blend Electrolytes: Role of Lattice Energy of Magnesium Salts on EDLC Performance. Molecules.

[51] Svensson A.M., Valøen L.O., Tunold R. (2005). Modeling of the impedance response of porous metal hydride electrodes. Electrochim. Acta.

[52] Brza M., Aziz S., Anuar H., Alshehri S., Ali F., Ahamad T., Hadi J. (2021). Characteristics of a Plasticized PVA-Based Polymer Electrolyte Membrane and H^+^ Conductor for an Electrical Double-Layer Capacitor: Structural, Morphological, and Ion Transport Properties. Membranes.

[53] Polu A.R., Kumar R. (2011). Impedance Spectroscopy and FTIR Studies of PEG—Based Polymer Electrolytes. E-J. Chem..

[54] Aziz S.B., Abdullah O.G., Rasheed M.A. (2017). Structural and electrical characteristics of PVA:NaTf based solid polymer electrolytes: Role of lattice energy of salts on electrical DC conductivity. J. Mater. Sci. Mater. Electron..

[55] Zulkifli A.M., Aqilah Mat Said N.I., Aziz S.B., Ali Dannoun E.M., Hisham S., Shah S., Bakar A.A., Zainal Z.H., Tajuddin H.A., Hadi J.M. (2020). Characteristics of Dye-Sensitized Solar Cell Assembled from Modified Chitosan-Based Gel Polymer Electrolytes Incorporated with Potassium Iodide. Molecules.

[56] Pradhan D.K., Samantaray B.K., Choudhary R.N.P., Karan N.K., Thomas R., Katiyar R.S. (2010). Effect of plasticizer on structural and electrical properties of nanocomposite solid polymer electrolytes. Ionics.

[57] Aziz S., Dannoun E., Hamsan M., Ghareeb H., Nofal M., Karim W., Asnawi A., Hadi J., Kadir M. (2021). A Polymer Blend Electrolyte Based on CS with Enhanced Ion Transport and Electrochemical Properties for Electrical Double Layer Capacitor Applications. Polymers.

[58] Aziz S.B., Asnawi A.S., Abdulwahid R.T., Ghareeb H.O., Alshehri S.M., Ahamad T., Hadi J.M., Kadir M. (2021). Design of potassium ion conducting PVA based polymer electrolyte with improved ion transport properties for EDLC device application. J. Mater. Res. Technol..

[59] Aziz S., Asnawi A., Kadir M., Alshehri S., Ahamad T., Yusof Y., Hadi J. (2021). Structural, Electrical and Electrochemical Properties of Glycerolized Biopolymers Based on Chitosan (CS): Methylcellulose (MC) for Energy Storage Application. Polymers.

[60] Nofal M.M., Hadi J.M., Aziz S.B., Brza M.A., Asnawi A.S., Dannoun E., Abdullah A.M., Kadir M.F. (2021). A Study of Methyl-cellulose Based Polymer Electrolyte Impregnated with Potassium Ion Conducting Carrier: Impedance, EEC Modeling, FTIR, Dielectric, and Device Characteristics. Materials.

[61] Hadi J.M., Aziz S.B., Kadir M., El-Badry Y.A., Ahamad T., Hussein E.E., Asnawi A.S., Abdullah R.M., Alshehri S.M. (2021). Design of Plasticized Proton Conducting Chitosan:Dextran Based Biopolymer Blend Electrolytes for EDLC Application: Structural, Impedance and Electrochemical Studies. Arab. J. Chem..

[62] Hadi J.M., Aziz S.B., Saeed S.R., Brza M.A., Abdulwahid R.T., Hamsan M.H., Abdullah R.M., Kadir M.F.Z., Muzakir S.K. (2020). Investigation of Ion Transport Parameters and Electrochemical Performance of Plasticized Biocompatible Chitosan-Based Proton Conducting Polymer Composite Electrolytes. Membranes.

[63] Kumari V.S., Basha S.K., Sudha P.N. (2011). Physicochemical and morphological evaluation of chitosan/poly(vinyl alcohol)/methylcellulose chemically cross-linked ternary blends. Polym. Bull..

[64] Negim E.S., Rakhmetullayeva R.K., Yeligbayeva G.Z., Urkimbaeva P.I., Primzharova S.T., Kaldybekov D.B., Khatib J.M., Mun G.A., Craig W. (2014). Improving biodegradability of polyvinyl alcohol/starch blend films for packaging applications. Int. J. Basic Appl. Sci..

[65] Choo K., Ching Y.C., Chuah C.H., Julai S., Liou N.-S. (2016). Preparation and Characterization of Polyvinyl Alcohol-Chitosan Composite Films Reinforced with Cellulose Nanofiber. Materials.

[66] Abdullah O., Aziz S.B., Rasheed M.A. (2016). Structural and optical characterization of PVA: KMnO_4_ based solid polymer electrolyte. Results Phys..

[67] Tamilselvi P., Hema M. (2016). Structural, thermal, vibrational, and electrochemical behavior of lithium ion conducting solid polymer electrolyte based on poly(vinyl alcohol)/poly(vinylidene fluoride) blend. Polym. Sci. Ser. A.

[68] Aziz S.B., Brza M., Mohamed P.A., Kadir M., Hamsan M., Abdulwahid R.T., Woo H. (2019). Increase of metallic silver nanoparticles in Chitosan:AgNt based polymer electrolytes incorporated with alumina filler. Results Phys..

[69] Aziz S.B. (2016). Role of dielectric constant on ion transport: Reformulated Arrhenius equation. Adv. Mater. Sci. Eng..

[70] Teo L.P., Buraidah M.H., Nor A.F.M., Majid S.R. (2012). Conductivity and dielectric studies of Li_2_SnO_3_. Ionics.

[71] Dannoun E.M., Aziz S.B., Kadir M., Brza M., Nofal M.M., Hadi J.M., Al-Saeedi S.I., Abdulwahid R.T. (2022). The Study of Impedance, Ion Transport Properties, EEC Modeling, Dielectric and Electrochemical Characteristics of Plasticized Proton Conducting PVA Based Electrolytes. J. Mater. Res. Technol..

[72] Awasthi P., Das S. (2019). Reduced electrode polarization at electrode and analyte interface in impedance spectroscopy using carbon paste and paper. Rev. Sci. Instrum..

[73] Khiar A.S.A., Anuar M., Parid M.M. (2016). Effect of 1-Ethyl-3-Methylimidazolium Nitrate on the Electrical Properties of Starch/Chitosan Blend Polymer Electrolyte. Mater. Sci. Forum.

[74] Hadi J.M., Aziz S.B., Mustafa M.S., Hamsan M.H., Abdulwahid R.T., Kadir M.F.Z., Ghareeb H.O. (2020). Role of nano-capacitor on dielectric constant enhancement in PEO:NH_4_SCN:xCeO_2_ polymer nano-composites: Electrical and electrochemical properties. J. Mater. Res. Technol..

[75] Abid Z., Hakiki A., Boukoussa B., Launay F., Hamaizi H., Bengueddach A., Hamacha R. (2019). Preparation of highly hydrophilic PVA/SBA-15 composite materials and their adsorption behavior toward cationic dye: Effect of PVA content. J. Mater. Sci..

[76] Arya A., Sharma A.L. (2018). Effect of salt concentration on dielectric properties of Li-ion conducting blend polymer electrolytes. J. Mater. Sci. Mater. Electron..

[77] Ayesh A.S. (2010). Electrical and optical characterization of PMMA doped with Y_0.0025_Si_0.025_Ba_0.9725_ (Ti_(0.9)_Sn_0.1_)O_3_ ceramic. Chin. J. Polym. Sci..

[78] Morsi M.A., Oraby A.H., Elshahawy A.G., Abd El-Hady R.M. (2019). Preparation, structural analysis, morphological investigation and electrical properties of gold nanoparticles filled polyvinyl alcohol/carboxymethyl cellulose blend. J. Mater. Res. Technol..

[79] Richert R., Richert R. (2002). The modulus of dielectric and conductive materials and its modification by high electric fields. J. Non-Cryst. Solids.

[80] Brza M.A. (2021). Electrochemical Impedance Spectroscopy as a Novel Approach to Investigate the Influence of Metal Complexes on Electrical Properties of Poly(vinyl alcohol) (PVA) Composites. Int. J. Electrochem. Sci..

[81] Nofal M.M., Aziz S.B., Hadi J.M., Abdulwahid R.T., Dannoun E.M.A., Marif A.S., Al-Zangana S., Zafar Q., Brza M.A., Kadir M.F.Z. (2020). Synthesis of Porous Proton Ion Conducting Solid Polymer Blend Electrolytes Based on PVA: CS Polymers: Structural, Morphological and Electrochemical Properties. Materials.

[82] Baskaran R., Selvasekarapandian S., Kuwata N., Kawamura J., Hattori T. (2006). ac impedance, DSC and FT-IR investigations on (x)PVAc–(1−x)PVdF blends with LiClO_4_. Mater. Chem. Phys..

[83] Belattar J., Graça M.P.F., Costa L.C., Achour M.E., Brosseau C. (2010). Electric modulus-based analysis of the dielectric relaxation in carbon black loaded polymer composites. J. Appl. Phys..

[84] Pradhan D.K., Choudhary R.N.P., Samantaray B.K. (2008). Studies of dielectric relaxation and AC conductivity behavior of plasti-cized polymer nanocomposite electrolytes. Int. J. Electrochem. Sci..

[85] Sengwa R.J., Choudhary S., Sankhla S. (2008). Low frequency dielectric relaxation processes and ionic conductivity of montmorillonite clay nanoparticles colloidal suspension in poly(vinyl pyrrolidone)−ethylene glycol blends. Express Polym. Lett..

[86] Amudha S., Suthanthiraraj S.A. (2015). Silver Ion Conducting Characteristics of a Polyethylene Oxide-based Composite Polymer Electrolyte And Application In Solid State Batteries. Adv. Mater. Lett..

[87] Polu A.R., Kumar R. (2012). Ionic Conductivity and Discharge Characteristic Studies of PVA-Mg(CH_3_COO)_2_ Solid Polymer Electrolytes. Int. J. Polym. Mater..

[88] Priya S.S., Karthika M., Selvasekarapandian S., Manjuladevi R. (2018). Preparation and characterization of polymer electrolyte based on biopolymer I-Carrageenan with magnesium nitrate. Solid State Ionics.

[89] Ponraj T., Ramalingam A., Selvasekarapandian S., Srikumar S.R., Manjuladevi R. (2020). Plasticized solid polymer electrolyte based on triblock copolymer poly(vinylidene chloride-co-acrylonitrile-co-methyl methacrylate) for magnesium ion batteries. Polym. Bull..

[90] Mokhtar M., Majlan E.H., Ahmad A., Tasirin S.M., Daud W.R.W. (2018). Effect of ZnO Filler on PVA-Alkaline Solid Polymer Electrolyte for Aluminum-Air Battery Applications. J. Electrochem. Soc..

[91] Aziz S.B., Nofal M.M., Abdulwahid R.T., Kadir M.F.Z., Hadi J.M., Hessien M.M., Kareem W.O., Dannoun E.M.A., Saeed S.R. (2021). Impedance, FTIR and transport properties of plasticized proton conducting biopolymer electrolyte based on chitosan for electrochemical device application. Results Phys..

[92] Monisha S., Mathavan T., Selvasekarapandian S., Benial A.M.F., Latha M.P. (2017). Preparation and characterization of cellulose acetate and lithium nitrate for advanced electrochemical devices. Ionics.

